# Elevated light intensity compensates for nitrogen deficiency during chrysanthemum growth by improving water and nitrogen use efficiency

**DOI:** 10.1038/s41598-022-14163-4

**Published:** 2022-06-15

**Authors:** Sara Esmaeili, Sasan Aliniaeifard, Shirin Dianati Daylami, Soheil Karimi, Aida Shomali, Fardad Didaran, Arkadiusz Telesiński, Edyta Sierka, Hazem M. Kalaji

**Affiliations:** 1 Photosynthesis Laboratory, Department of Horticulture, Aburaihan Campus, University of Tehran, Tehran, 33916-53755 Iran; 2 Department of Horticulture, Aburaihan Campus, University of Tehran, Tehran, 33916-53755 Iran; 3 Department of Bioengineering, West Pomeranian University of Technology in Szczecin, 17 Słowackiego Street, 71-434 Szczecin, Poland; 4 Institute of Biology, Biotechnology and Environmental Protection, Faculty of Natural Sciences, University of Silesia in Katowice, 28 Jagiellonska, 40-032 Katowice, Poland; 5 Department of Plant Physiology, Institute of Biology, Warsaw University of Life Sciences—SGGW, 02-787 Warsaw, Poland

## Abstract

Identifying environmental factors that improve plant growth and development under nitrogen (N) constraint is essential for sustainable greenhouse production. In the present study, the role of light intensity and N concentrations on the biomass partitioning and physiology of chrysanthemum was investigated. Four light intensities [75, 150, 300, and 600 µmol m^−2^ s^−1^ photosynthetic photon flux density (PPFD)] and three N concentrations (5, 10, and 15 mM N L^−1^) were used. Vegetative and generative growth traits were improved by increase in PPFD and N concentration. High N supply reduced stomatal size and g_s_ in plants under lowest PPFD. Under low PPFD, the share of biomass allocated to leaves and stem was higher than that of flower and roots while in plants grown under high PPFD, the share of biomass allocated to flower and root outweighed that of allocated to leaves and stem. As well, positive effects of high PPFD on chlorophyll content, photosynthesis, water use efficiency (WUE), Nitrogen use efficiency (NUE) were observed in N-deficient plants. Furthermore, photosynthetic functionality improved by raise in PPFD. In conclusion, high PPFD reduced the adverse effects of N deficiency by improving photosynthesis and stomatal functionality, NUE, WUE, and directing biomass partitioning toward the floral organs.

## Introduction

Nitrogen (N) is one of the most important nutritional elements for plants and the main constituent of many essential molecules such as chlorophyll, amino acids, and nucleic acids^1–3^. It also plays a crucial role as signaling molecule in form of nitric oxide under stress condition^4^. The deficiency of N is often a limiting factor for plant growth and development^5,6^. Modern agriculture approaches to maximize the efficiency of resources. N is known as the most consumed fertilizer used to increase crop production^7^. However, more than half of the N that used in agriculture is lost through leaching and evaporation^8^. Excessive use of chemical fertilizers, especially N fertilizers, increases the cost of crop production and greenhouse gas emissions, as well as soil and groundwater pollution^9,10^. Therefore, optimization of crop production under nitrogen restriction conditions is a major challenge in agriculture^11^.

Light is the primary energy source for photosynthesis. Light intensity, quality (spectrum), and duration (photoperiod) influence plant growth and development^12,13^. The main absorption spectra of the chlorophyll pigments are in the range of blue and red light, therefore, these two spectra are mainly used for plant production in controlled environments^13^. Besides light quality, the intensity of the light also exerts a broad range of physiological effects on plant growth and performance^14–16^, including, N uptake and allocation^17^.

Light is among the environmental factors that affect the absorption and reduction of nitrogen in plants^18^. Light intensity and N concentration have been shown to play a crucial role in the N uptake and leaf N content of plants. It was shown that raise in light intensity increases the activity of enzymes involved in N metabolism^19^. In addition provision of adequate irradiance increases the N assimilation in plant leaves by providing enough energy for CO_2_ fixation^20^.

Today, there is a high tendency to produce ornamental plants in greenhouses and controlled environments and there is a need to determine the desirable PPFD for plant growth in an approach with the highest resource use efficiency. The nature and extent of plant response to light intensity are dependent on the N status of the plant and vice versa^21,22^. Therefore, optimizing the N concentration of nutrient solution based on light intensity can be effective in achieving the best growth and yield of ornamental plants. To our knowledge, so far, no study has examined the effect of increasing light intensity as an effective strategy and environmentally compatible to reduce growth defects under N deficient conditions and increase nitrogen use efficiency (NUE).

Chrysanthemum is one of the most popular ornamental plants, which ranked second place after roses in the ornamental industry worldwide^23^. Given that, both light and N are the most important limiting factors for the growth and yield of chrysanthemum. In this study, we hypothesized that light intensity may affect N metabolism and an increase in light intensity may compensate for N deficiency.

Here, we investigated the changes of the morphological and physiological characteristics, as well as, biomass partitioning of chrysanthemum in response to different combinations of light intensity and N concentrations of the nutrient solution. This study provides valuable insights into the interactive regulation of light intensity and N supply to improve the quality of chrysanthemum production.

## Results

### N constraint intervention in chlorophyll fluorescence is more pronounced in old leaves rather than young leaves under different PPFDs

**Figure 1:**
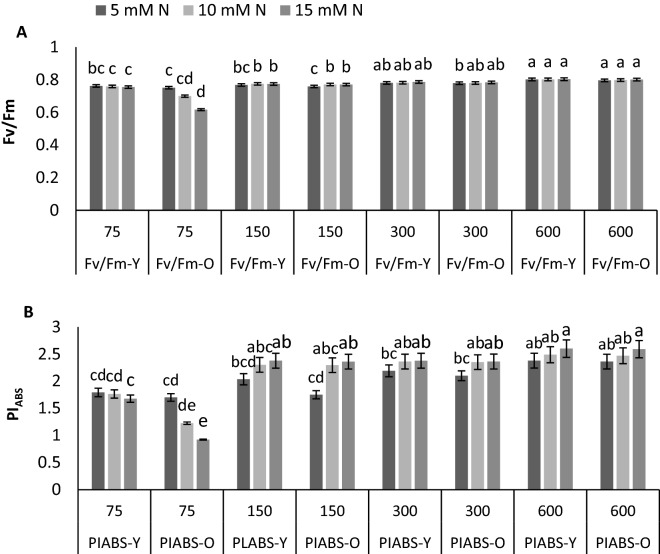
Quantum yield of PSII (F_V_/F_M_) (**A**), and performance index per absorbed light (PI_ABS_) (**B**) of young (Y) and old (O) leaves of chrysanthemum plants grown under different light intensits (75, 150, 300, and 600 µmol m^−2^ s^−1^) and nitrogen (N) concentrations (5, 10, and 15 mM N) at 70 days of cultivation. Vertical bars are means ± SD. Within each graph, interactive effects of light intensity and nitrogen concentration are shown. Different letters (a–d) denote a significant difference between treaments (P < 0.05).

To evaluate the effects of different N supplies under various PPFDs on photosynthesis functionality, F_V_/F_M_ of chrysanthemum young and old leaves was measured. An increase in F_V_/F_M_ was observed by the increase in PPFD while the effect of N concentration was negligible. However, the effect of N concentration on F_V_/F_M_ on old leaves of plants grown under 75 µmol m^−2^ s^−1^ was considerable compared to old leaves of plants grown under other PPFDs. Under low light intensity, F_V_/F_M_ decreased by 6% and 17% by an increase in N concentration from 5 to 10 and 15 mM N L^−1^, respectively (Fig. 1A). PI_ABS_ was increased by raise in PPFD but the effect of N concentration was negligible on young leaves. In old leaves, however, by the increase in N concentration from 5 to 10 and 15 mM N L^−1^, 27% and 45% decline displayed by plants grown under 75 µmol m^−2^ s^−1^. In contrast, the old leaves of plants grown under 150, 300, and 600 µmol m^−2^ s^−1^, showed higher PI_ABS_, when fed by 10 and 15 mM N L^−1^ compared to that of plants under similar PPFD but fed by 5 mM N L^−1^ (Fig. 1B). Considering the effects of PPFD on PI_ABS_ of plants under N constraint revealed that, increase in PPFD enhanced PI_ABS_ of plants indicated by 13%, 22%, and 32% increase in young leaves and 2%, 23%, and 38% increase in old leaves by raise in PPFD from 75 to 150, 300 and 600 µmol m^−2^ s^−1^, respectively (Fig. 1B).

### Chlorophyll concentration of leaves were strongly associated with nitrogen content under high PPFD but not under low PPFD

**Figure 2:**
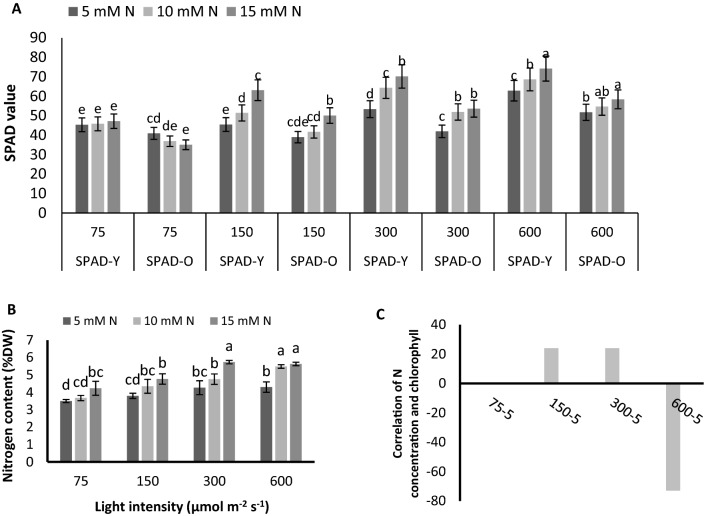
Chlorophyll content of the leaves (**A**), Nitrogen content of leaves (**B**), and the correlation between N and chlorophyll cntent (**C**) of the leaves of chrysanthemum plants grown under different light intensits (75, 150, 300, and 600 µmol m^−2^ s^−1^) and nitrogen (N) concentrations (5, 10, and 15 mM N) at 70 days of cultivation. Vertical bars are means ± SD. Within each graph, interactive effects of light intensity and nitrogen concentration are shown. Different letters (a–d) denote a significant difference between treaments (P < 0.05).

Chlorophyll content increased stepwise by the increase in both PPFD and N concentration (Fig. 2A). By increase in N concentration from 5 to 10 and 15 mM N L^−1^ , the increase in chlorophyll content was 24% and 38% under 75 µmol m^−2^ s^−1^, 10% and 39% under 150 µmol m^−2^ s^−1^, 11% and 27% under 300 µmol m^−2^ s^−1^ and 19% and 30% under 600 µmol m^−2^ s^−1^ (Supplementary Fig. 1). Furthermore, under N constraint (5 mM N L^−1^), an increase in PPFD compensated for chlorophyll synthesis. By raise in PPFD form 75 to 150, 300 and 600 µmol m^−2^ s^−1^, 40%, 60% and 76% increases were observed in chlorophyll content (Fig. 2A). Comparing the chlorophyll content of young and old leaves revealed that, regardless of N concentration and PPFD, the chlorophyll content of old leaves was smaller than young leaves. Moreover, in both young and old leaves increase in PPFD increased the chlorophyll content of the leaves regardless of N concentration. In young leaves, a raise in N concentration increased the chlorophyll content of plants under each PPFDs (Fig. 2A). In old leaves, however, a contrasting trend was observed in the chlorophyll content of leaves in plants grown under 75 µmol m^−2^ s^−1^ and plants grown under higher PPFDs. The chlorophyll content of the old leaves of plants under PPFDs higher than 75 µmol m^−2^ s^−1^ increased by raise in N concentration, while its content in plants grown under 75 µmol m^−2^ s^−1^ decreased by an increase in N concentration (Fig. 2A). The nitrogen content of the leaves increased by raise in PPFD in all N concentrations (Fig. 2B). The highest N content was observed in plants fed by 15 mM N L^−1^ under 300 µmol m^−2^ s^−1^ and plants fed by 10 and 15 mM N L^−1^ under 600 µmol m^−2^ s^−1^. N content in plants grown under 300 and 600 µmol m^−2^ s^−1^ and 15 mM N L^−1^ was 33% higher than that of plants under the same N concentration but exposed to 75 µmol m^−2^ s ^−1^ (Fig. 2B). When plants were fed by 10 mM N L^−1^, the N content of leaves was 16%, 29%, and 49% higher in plants grown under 150, 300, and 600 µmol m^−2^ s^−1^ compared to the plants fed by 5 mM N L^−1^. In plants fed by 5 mM N L^−1^, the compensatory role of PPFD was conceived since 8%, 22%, and 31% increase in N content of leaves obtained by an increase in PPFD form 150 to 300 and 600 µmol m^−2^ s^−1^ (Fig. 2B).

Moreover, the correlation between N content of the leaves with chlorophyll content was negligible in plants exposed to 75 µmol m^−2^ s^−1^ but the chlorophyll conent of plants grown under 150 µmol m^−2^ s^−1^ and 300 µmol m^−2^ s^−1^ was positively correlated with N content of the leaves. Nevertheless, when the PPFD reached 600 µmol m^−2^ s^−1^ the correlation droped drastically (Fig. 2C; Supplementary Figs. 2, 3).

### Stomatal aperture and gas exchange enhanced by raise in PPFD but not affected by N unless the PPFD was low

**Figure 3:**
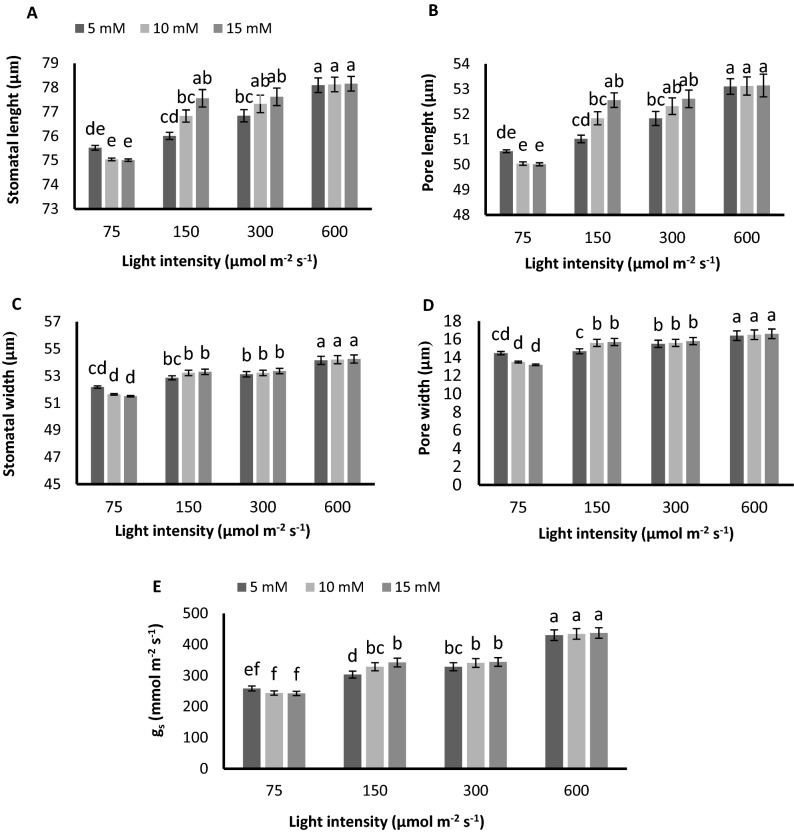
Stomatal lenght (**A**), stomatal pore lenght (**B**), stomatal width (**C**), stomatal pore width (**D**), and stomatal conductance (g_s_) (**E**) of chrysanthemum plants grown under different light intensits (75, 150, 300, and 600 µmol m^−2^ s^−1^) and nitrogen (N) concentrations (5, 10, and 15 mM N) at 70 days of cultivation. Vertical bars are means ± SD. Within each graph, interactive effects of light intensity and nitrogen concentration are shown. Different letters (a–d) denote a significant difference between treaments (P < 0.05).

Stomatal traits showed contrasting responses to N concentration when exposed to different light intensities. In plants grown under 75 µmol m^−2^ s^−1^, stomatal aperture, stomatal length and width and stomatal conductance decreased by the increase in N concentration (Fig. 3A-E). However, the opposing trend was observed when plants were exposed to PPFDs higher than 75 µmol m^−2^ s^−1^. In plants grown under 150 and 300 µmol m^−2^ s^−1^, stomatal length and stomatal pore length increased by the increase in N concentration while under 600 µmol m^−2^ s^−1^, increase in N concentration didn’t affect stomatal traits. A negligible increase in stomatal width, stomatal pore width, and stomatal conductance was displayed by the increase in N concentration in plants grown under 150 µmol m^−2^ s^−1^ (Fig. 3A–E).

### Light intensity affect the accumulation and partitioning of biomass under contrasting nitrogen regimes

**Figure 4:**
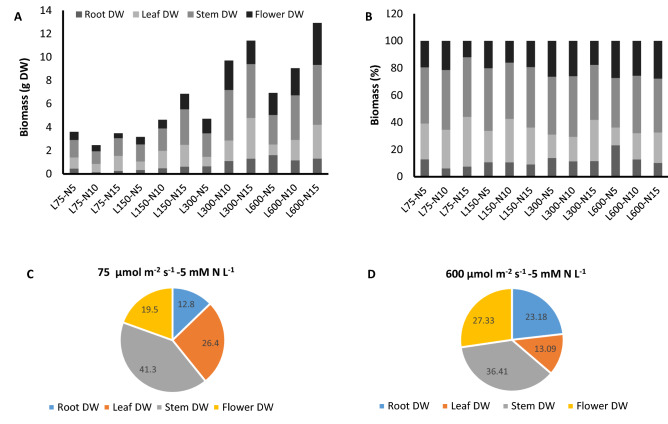
Total biomass (**A**), biomass partitioning among different organs (**B**), biomass partitioning among different organs in plants grown under 75 µmol m^−2^ s^−1^ and 5 mM N (**C**) and biomass partitioning among different organs in plants grown under 600 µmol m^−2^ s^−1^ and 5 mM N (**D**) of chrysanthemum grown under different light intensits (75, 150, 300, and 600 µmol m^−2^ s^−1^) and nitrogen (N) concentrations (5, 10, and 15 mM N) at 70 days of cultivation. Vertical bars are means ± SD. Within each graph, interactive effects of light intensity and nitrogen concentration are shown. Different letters (a–d) denote a significant difference between treaments (P < 0.05).

The total biomass was affected by PPFD and N concentration. By increase in N concentration, a stepwise elevation was observed in total biomass under each PPFD except for 75 µmol m^−2^ s^−1^. Regardless of N concentration, total biomass increased by raise in PPFD, except the biomass of plants under 5 mM N L^−1^ that did not differ under 75 or 150 µmol m^−2^ s^−1^ (Fig. 4A). Under N constraint, PPFD could compensate for N limitation for biomass accumulation. In plants fed by 5 mM N L^−1^ total biomass of plants grown under 600 µmol m^−2^ s^−1^ was twofold of the plants grown under 75 and 150 µmol m^−2^ s^−1^ and 1.5-fold of the total biomass of plants under 300 µmol m^−2^ s^−1^ (Fig. 4A).

Partitioning of biomass to different plant organs was also affected by both light intensity and N concentration (Fig. 4B). Root biomass showed 52% and 41% decline by the increase in N concentration from 5 to 10 and 15 under 75 µmol m^−2^ s^−1^ (Fig. 4B). Increasing PPFD form 75 to 150 µmol m^−2^ s^−1^, partitioning of biomass to root decreased by 0.8% and 12% respectively by increase in N concentration from 5 to 10 and 15. Same as the plants exposed to 150 µmol m^−2^ s^−1^, by the increase in N concentration form 5 to 10 and 15, respectively 17% and 16% increase in the partitioning of biomass to the roots were detected in plants exposed to 300 µmol m^−2^ s^−1^ (Fig. 4B). However, the reduction in the partitioning of biomass to root was more drastic under 600 µmol m^−2^ s^−1^ in plants fed by 10 and 15 mM N L^−1^ compared to the plants fed by 5 mM N L^−1^, indicated by 45% and 55% reduction in root biomass, suggesting the compensatory effect of high PPFD on the partitioning of biomass to root under N constraint (Fig. 4B).

Partitioning of biomass to leaves increased when N concentration raised from 5 to 10 and 15 mM N L^−1^ under all PPFDs. By increase in N concentration from 5 to 10 and 15 respectively, the increase in partitioning of biomass to leaves were recorded as 7% and 38% in plants under 75 µmol m^−2^ s^−1^, 38% and 17% in plant under 150 µmol m^−2^ s^−1^, 5% and 75% increase in plants under 300 µmol m^−2^ s^−1^, and 47% and 70% in plants under 600 µmol m^−2^ s^−1^ (Fig. 4B).

Partitioning of biomass to stem increased by raise in N concentration from 5 to 10 and 15 mM N L^−1^ by 6% in plants under 75 µmol m^−2^ s^−1^ and 16% and 9% in plants under 600 µmol m^−2^ s^−1^. In contrast, in plants grown under 150 and 300 µmol m^−2^ s^−1^, a small reduction was observed in biomass partitioning to stem indicated by 10% and 3% decrease respectively by raise in N concentration from 5 to 10 and 15 mM N L^−1^. A 5% decline in the partitioning of biomass to stem was also displayed by plants grown under 300 µmol m^−2^ s^−1^ and fed by 15 mM N L^−1^ compared to plants under the same PPFD but 5 mM N L^−1^ (Fig. 4B).

**Figure 5:**
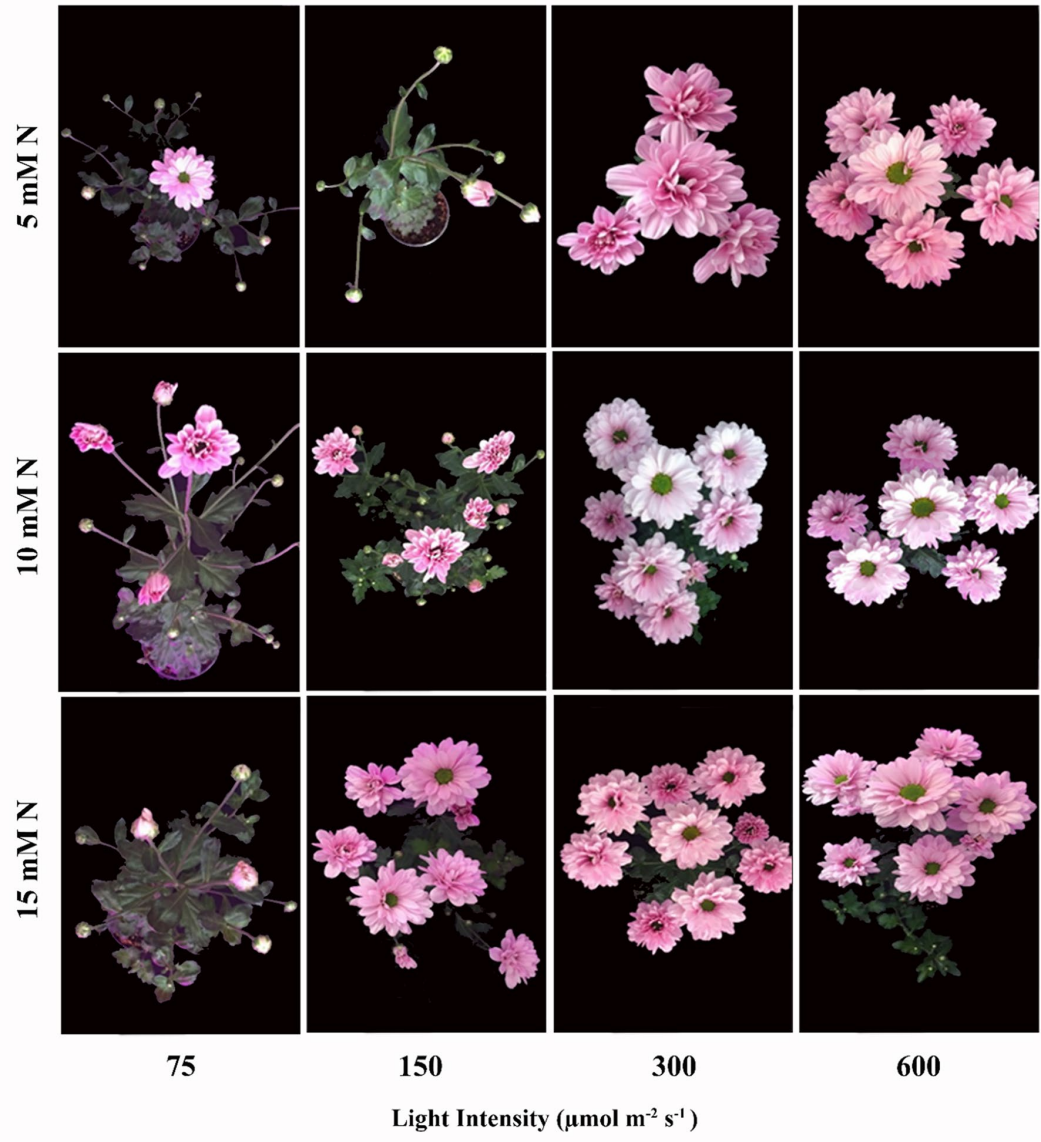
Interaction effect of light intensity and concentration of nitrogen (N) on the flowering of chrysanthemum. Plants were exposed to different concentrations of nitrogen (N) (5, 10, and 15 mM N) and light intensities (75, 150, 300, and 600 µmol m^−2^ s^−1^).

Partitioning of biomass to flower was also affected by N concentration and PPFD. In plants grown under 75 µmol m^−2^ s^−1^, a 10% increase in the partitioning of biomass to flower was observed by raise in N concentration from 5 to 10 mM N L^−1^. Whereas, an increase in N concentration from 5 to 15 mM N L^−1^ resulted in a 38% decline in biomass partitioning to flower (Figs. 4B, 5). Increase in N concentration from 5 to 10 and 15 mM N L^−1^ cut down on partitioning of biomass to flowers by 20% and 3% under 150 µmol m^−2^ s^−1^. As well, 33% increase in biomass partitioning to flowers observed in plants under 300 µmol m^−2^ s^−1^ when N concentration increase from 5 to 15 mM N L^−1^. In plants grown under 600 µmol m^−2^ s^−1^, an increase in N concentration from 5 to 10 mM N L^−1^ reduced biomass partitioning to flower (Fig. 4B).

Overall, comparing the partitioning of biomass to different organs revealed that, under low PPFD, the share of biomass allocated to leaves (26%) and stem (41%) was higher than that of flower (19%) and root (13%), while, in plants grown under high PPFD, the share of biomass allocated to flower (27%) and root (23%) increased at the expense of biomass accumulation in leaves (23%) and stem (13%) (Fig. 4C,D).

### WUE decreased by higher N concentration under low PPFD while increased by higher N concentration under high PPFD

**Figure 6:**
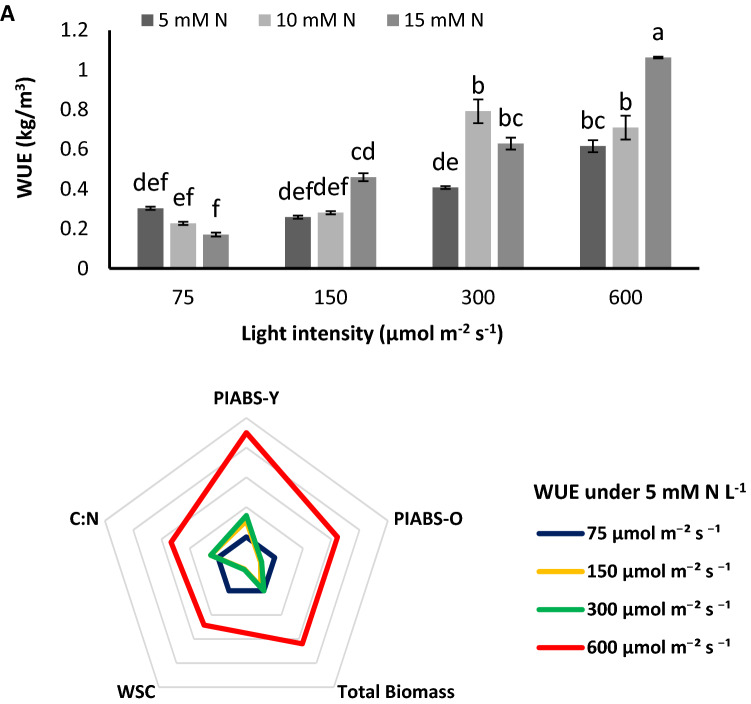
Water use efficiency (WUE) (**A**) of chrysanthemum plants grown under different light intensits (75, 150, 300, and 600 µmol m^−2^ s^−1^) and nitrogen (N) concentrations (5, 10, and 15 mM N) at 70 days of cultivation. The correlation between WUE with performance index per absorbed light (PI_ABS_), total biomass, WSC, and C:N ration of plants fed by 5 mM N with under differenty light intensities (150, 300, and 600 µmol m^−2^ s^−1^) compared to that of plants under 75 µmol m^−2^ s^−1^ (**B**). Vertical bars are means ± SD. Within each graph, interactive effects of light intensity and nitrogen concentration are shown. Different letters (a–d) denote a significant difference between treaments (P < 0.05).

WUE was affected by N concentration but came to contradictory effects in plants grown under 75 µmol m^−2^ s^−1^ compared to plants grown under 150, 300 and 600 µmol m^−2^ s^−1^ (Fig. 6A). Under 75 µmol m^−2^ s^−1^, by increase in N concentration form 5 to 10 and 15 mM N L^−1^, 25 and 43% reduction in WUE was observed, conversely, 8% and 77% increase under 150 µmol m^−2^ s^−1^, 94% and 54% increase under 300 µmol m^−2^ s^−1^ and 15% and 72% increase under 600 µmol m^−2^ s^−1^ were detected in WUE of plants by the increase in the concentration of N from 5 to 10 and 15 mM N L^−1^, respectively (Fig. 6A). Considering the effect of PPFD on WUE of plants grown under N constraint revealed that increase in PPFD from 75 to 150 µmol m^−2^ s^−1^ did not affect the WUE of plants and when PPFD was raised to 300 µmol m^−2^ s^−1^ a negligible increase was observed, whereas when PPFD reached to 600 µmol m^−2^ s^−1^, WUE of plants increased to twofold of WUE of plants under 75 µmol m^−2^ s^−1^ (Fig. 6A). The correlation of total biomass with WUE also increased by the increase in PPFD under N constraint, indicating that the water uptaken by the plant were efficiently utilized for biomass accumulation. The correlation of PI_ABS_ with WUE was more profound on young leaves compared to old leaves; since the correlation was strongly negative under low PPFD and raised sharply under high PPFD in young leaves, while in old leaves the increase in correlation by raise in PPFD was slighter than that of young leaves (Fig. 6B). In addition, under N constraint, the correlation between PI_ABS_ and WUE was affected by PPFD. WUE was negatively correlated with PI_ABS_ of young and old leaves under N constraint but an increase in PPFD strengthen the correlation and increase it from − 76 to − 50% in young and old leaves under 75 µmol m^−2^ s^−1^ to 99% and 61% under 600 µmol m^−2^ s^−1^ (Fig. 6B). The effect of PPFD on increasing WUE under N constraint was more pronounced than that of old leaves. Moreover, according to correlation results, WUE and PI_ABS_ are more closely associated in young leaves compared to the old leaves (Fig. 6B; Supplementary Figs. 2, 3).

### Carbohydrate level depleted by increasing N concentration of nutrient solution under low PPFD, while accumulated by exposure to higher PPFDs

**Figure 7:**
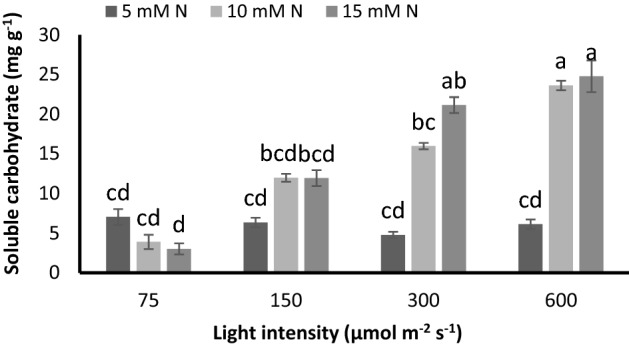
Water soluble carbohydrates (WSC) of chrysanthemum plants grown under different light intensits (75, 150, 300, and 600 µmol m^−2^ s^−1^) and nitrogen (N) concentrations (5, 10, and 15 mM N) at 70 days of cultivation. Vertical bars are means ± SD. Within each graph, interactive effects of light intensity and nitrogen concentration are shown. Different letters (a–d) denote a significant difference between treaments (P < 0.05).

A contrasting trend was observed on the effect of N concentration on soluble carbohydrates under different light intensities (Fig. 7). In plants grown under 75 µmol m^−2^ s^−1^ the carbohydrate decreased by 44% and 57% by an increase in N concentration from 5 to 10 and 15 mM N L^−1^, respectively (Fig. 7). In contrast, in plants grown under PPFDs higher than 75 µmol m^−2^ s^−1^, the carbohydrate content increased by raise in N concentration. Under 150 µmol m^−2^ s^−1^, 88% increase in carbohydrate was detected in plants fed by 10 and 15 mM N L^−1^ compared to 5 mM N L^−1^ (Fig. 7). Moreover, by raise in N concentrations from 5 to 10 and 15 mM N L^−1^, 2- and 3-fold increases were observed in the carbohydrate of plants grown under 300 and 600 µmol m^−2^ s^−1^ (Fig. 7). Regardless of N concentration, a raise in PPFD increased soluble carbohydrates. When plants were fed by 5 mM N L^−1^, 9%, 32%, and 12% reduction were observed in carbohydrates by the increase in PPFD from 75 to 150, 300 and 600 µmol m^−2^ s^−1^. However, in plants fed by 10 mM N L^−1^, raise in PPFD from 75 to 150, 300 and 600 µmol m^−2^ s^−1^ resulted in 2-, 3- and 5-fold and under 15 mM N L^−1^ 2-, 5- and sevenfold increase in carbohydrate contents were observed by the increase in PPFD from 75 to 150, 300 and 600 µmol m^−2^ s^−1^, suggesting a positive role of PPFD on carbohydrate synthesis (Fig. 7). Our data also revealed that a negative correlation existed between WUE and carbohydrate under N constraint and low light (− 70%) and a raise in PPFD ameliorated the negative correlation, since under 600 µmol m^−2^ s^−1^ and N constraint, 61% correlation was detected between WUE and carbohydrate (Fig. 6B; Supplementary Figs. 2, 3).

### NUE decreased by higher N concentration while increased by higher PPFDs

**Figure 8:**
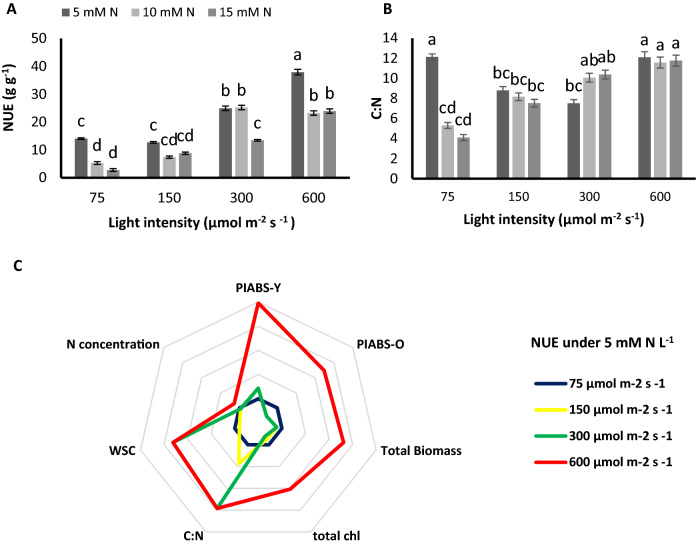
NUE of leaves (**A**) C:N ratio (**B**), of chrysanthemum plants grown under different light intensits (75, 150, 300, and 600 µmol m^−2^ s^−1^) and nitrogen (N) concentrations (5, 10, and 15 mM N) at 70 days of cultivation. The correlation between NUE of plants fed by 5 mM N with performance index per absorbed light (PI_ABS_), total biomass, total chlorophyll (chl), C:N ration, water soluble carbohydrates (WSC), and nitrogen concentration under differenty light intensities (150, 300, and 600 µmol m^−2^ s^−1^) compared to that of plants under 75 µmol m^−2^ s^−1^ (**C**). Vertical bars are means ± SD. Within each graph, interactive effects of light intensity and nitrogen concentration are shown. Different letters (a–d) denote a significant difference between treaments (P < 0.05).

NUE was increased by raise in PPFD, while declined by the increase in N concentration under all PPFDs (Fig. 8A). The highest NUE was associated with plants fed by the lowest N concentration and grown under the highest light intensity. When plants were fed by 5 mM N L^−1^, NUE was increased by 77% and 169% by raise in PPFD from 75 to 300 and 600 µmol m^−2^ s^−1^ and remained unchanged in plants grown under 150 µmol m^−2^ s^−1^ (Fig. 8A). In other words, a raise in PPFD compensated for the adverse effects of N constraint on NUE since the highest PPFD was associated with the highest NUE in plants grown under 5 mM N L^−1^. Under 300 and 600 µmol m^−2^ s^−1^, NUE of plants fed by 10 mM N L^−1^ was threefold of NUE of plants grown under 75 and 150 µmol m^−2^ s^−1^. Under 150, 300, and 600 µmol m^−2^ s^−1^, when plants were fed by 15 mM N L^−1^, NUE was 2-, 3- and 7-fold of NUE of plants grown under 75 µmol m^−2^ s^−1^ (Fig. 8A).

C:N ratio was decreased by raise in N concentration from 5 to 10 and 15 mM N L^−1^ by 56% and 66% respectively, in plants under 75 µmol m^−2^ s^−1^ and reduced by 14% and 7% in plant under 150 µmol m^−2^ s^−1^. Under 300 µmol m^−2^ s^−1^ however, C:N ratio showed 34% and 38% increase by raise in N concentration from 5 to 10 and 15 mM N L^−1^ (Fig. 8B).

The relation between NUE and biomass accumulation was also affected by PPFD. Under N constraint (5 mM N L^−1^) NUE was negatively correlated (− 73%) with total biomass while, when PPFD was raised to 600 µmol m^−2^ s^−1^, a positive correlation (58%) was detected between NUE and total biomass (Fig. 8C). Chlorophyll content was also demonstrated a negative correlation (− 78%) with NUE under low light and N constraint, whereas, under 600 µmol m^−2^ s^−1^ and N constraint the negative correlation ameliorated to a 24% positive correlation (Fig. 8C; Supplementary Figs. 2, 3).

## Discussion

F_V_/F_M_ reflects the maximum photochemical quantum yield of PSII^24^. In this research, variation in F_V_/F_M_ under different treatments was negligible. However, the change in F_V_/F_M_ was more detectable in old leaves under high N concentration compared to young leaves, suggesting that the dependency of F_V_/F_M_ value to N concentration depends on leaf age and PPFD. The fact that plants respond to N supply by redistributing N from older leaves to the youngest can elaborate on the dependency of F_V_/F_M_ to leaf age^25^. Moreover, under the N constraint condition, the correlation between NUE and F_V_/F_M_ showed opposite trends by the increase in PPFD. Under N constraint and low PPFD, a strong positive correlation was detected between NUE and F_V_/F_M_ of young leaves, which reduced to a strongly negative correlation by raise in PPFD. Whereas, the correlation was strongly negative in old leaves under low PPFD and ameliorated to a small positive correlation under high PPFD. This finding may explain that under N constraint, old leaves invest more N for increasing photosynthesis efficiency compared to that of young leaves because young leaves invest N for other pathways like biomass accumulations or carbohydrate synthesis since the correlation of NUE with total biomass and carbohydrate was higher than the correlation between NUE and F_V_/F_M_ under high PPFD and N constraints.

PI_ABS_ is a delicate parameter derived from chlorophyll fluorescence that indicates the photochemical performance of photosynthesis^26–28^. The reduction in PI_ABS_ of old leaves under low PPFD by raise in N concentration indicates the decline in energy conversion ability and the photosynthesis apparatus activity, which is also portrayed by light curves of plants under different PPFDs and N concentrations (Supplementary Fig. 4). This reduction in energy conversion potential accounts for reduced NUE and WUE under low PPFD. This circumstance was accompanied by a reduction in soluble carbohydrates which is the output of the photosynthesis process. The correlation between PI_ABS_ with NUE and WUE was negative under low light and low N concentration, while under high PPFD a positive correlation was detected. This finding allows us to postulate that increase in PPFD compensates for N limitation by increasing PI_ABS_, denoting higher efficiency of energy conversion that further provides energy for N uptake and increases the requirements for water uptake.

The negative correlation between chlorophyll content and N content under high PPFDs may account for the increase in the use of N in other processes besides chlorophyll synthesis since by increase in PPFD the increase in the correlation between NUE and total chlorophyll was lower than the correlation between NUE and other parameters like biomass and C:N balance. This may explain, in part, the strong reduction in correlation between chlorophyll and N content under high PPFD. In this regard, previous reports also proposed the effect of PPFD on the allocation of N between photosynthetic and non-photosynthetic pools as well as different N allocation between Rubisco and light-harvesting^29,30^. Besides, our data demonstrated a compensative role of PPFD for chlorophyll synthesis under N constraint conditions despite the negative correlation maintained among the N content of the leaves and chlorophyll content. This discrepancy suggests that high PPFD tends to tune the balance between the use of N among different pathways, while under low PPFD the negligible correlation between N content with chlorophyll, biomass, and C:N ratio explains the lack of balance between partitioning of N to different pathways under N constraint. Furthermore, the close relationship between N metabolism and light signals can be the other explanation for the role of PPFD in compensation of N constraint since N uptake is regulated by shoot-borne light signals, has shown to up-regulate the expression of root nitrogen transporters and enhance N uptake eventually^31,32^. Also, an association between high light intensity, plant water uptake, transpiration and the uptake of soil nitrogen by roots were proposed which is governed by mass flow water movement^31^. Many studies have reported that chlorophyll a, chlorophyll b, and total chlorophyll content correlate strongly with leaf N concentration so that chlorophyll content is reduced with N deficiency^33^.

The fact that an increase in stomatal aperture and stomatal conductance was accompanied by an increase in WUE explains the increase in PI_ABS_ and the highest biomass accumulation observed under high PPFD. Under N constraint, this circumstance accounts for improved growth and physiological status of plants, proposing a compensative role of PPFD for N constraint. An increase in N concentration and PPFD positively affected the N content of the leaves. The same effect was also reported on *Lolium perenne* L.^34^. Light signaling within the root system may trigger N uptake through the mediation of root nitrogen transporters^31,32^, further consequence in the enhancement of plant water uptake, transpiration, and the acquisition of soil nitrogen by roots under high light condition^31^. The correlation between leaf N concentration and NUE was negative under the N constraint condition, however, a raise in PPFD weakened this negative correlation, suggesting enhancing the role of PPFD for NUE under N constraint condition.

The increase in total biomass by the rise in PPFD and N concentration in plants grown under PPFDs higher than 75 µmol m^−2^ s^−1^ can be explained by improve in dry and fresh weight of plant organs (Supplementary Fig. 5) as well as the number of leaves, number of flowers, and plant height (Supplementary Fig. 6), which were the result of improved photosynthetic traits along with elevation of NUE and WUE. Under N constraint the correlation between total biomass with NUE and WUE increased sharply by raise in PPFD. The increased efficiency of water and nitrogen use may account for the ameliorative effect of PPFD on biomass accumulation under N constraint. Root biomass increased by the increase in light intensity and N concentration, which is in agreement with previous reports^35^. Nevertheless, the share of root biomass in total biomass decreased by raise in N concentration at the expense of an increase in the partitioning of biomass to aerial organs indicated by an increase in biomass partitioning to leaves. The fact that partitioning of biomass to root was limited by raise in N concentrations can be elaborated by optimal partitioning theory which explains that the biomass is allocated to the organ of the plant that is exposed to the most limiting resource^36–38^. This partitioning strategy is thought to minimize the stress imposed by the limiting resource^39^. Contrary to N concentration, rise in PPFD has shown to increase the partitioning of biomass to root, which can be vindicated by increased carbon availability provided by higher photosynthesis functionality under high PPFDs^40^. Concurringly, our data revealed a higher C:N ratio under high PPFD. Moreover, the correlation between NUE and C:N ratio was increased by the increase in PPFD, suggesting a balance in the increase in carbon assimilation and N uptake. An increase in PI_ABS_ along with WUE led to elevated carbon assimilation. In order to N uptake keep pace with carbon assimilation to maintain the balance of C:N, root biomass increased to maximize N uptake under N constraint. This circumstance elaborates on the underlying mechanism through which PPFD compensated for N uptake under N constraint. Concurred with our results, the positive effect of PPFD on root biomass reported on lettuce^14^ and chrysanthemum^41^. Regardless of PPFD, increase in N concentration reduced the partitioning of biomass to flowers. However, in the same N concentration, a larger proportion of biomass is allocated to flowers by the increase in PPFD, which can be explained by the increase in the number of flowers displayed by increased PPFD and N concentration (Supplementary Fig. 6).

We observed that the adverse effects of nitrogen deficiency extended to the reproductive growth stage and eventually prolonged the flower emergence period and decreased the biomass partitioning to the flower under low PPFD The positive role of PPFD on the flower emergence period and the increasing number of flowers have been also declared in the previous study on chrysanthemum^41^. In addition, a strong genetic correlation between flowering date and NUE has been reported in wheat^42^. In the same line, our data denotes a close relationship between light intensity and NUE, which may in part explain the promoting effect of PPFD on flower emergence, regardless of N concentration, since under all N treatments the correlation between NUE and PFFD was high (R^2^ ≥ 98%). Moreover, the correlation between NUE and day to flowering was negative under high PPFD and positive under low PPFD, suggesting that, increase in NUE shortens day to flowering under high PPFD and remains the opposite effect under low PPFD (Supplementary Figs. 2, 3).

Under high PPFD, WUE improved by the increase in N concentration, which is in line with the results of a previous study on *Robinia pseudoacacia*^43^. However, according to our data, when PPFD was limited to 75 µmol m^−2^ s^−1^, WUE decreased by raise in N concentration. Consistently, in *Capsicum annum* L., the WUE of shaded plants was far lower than unshaded plants^44^. On the other hand, under N constraint, PPFD compensated for WUE under N deficiency. The compensative effect of PPFD on WUE under N constraint is portrayed by increased PI_ABS_, C:N ratio, and total biomass.

On contrary with the N content of the leaves, NUE decreased by an increase in N concentration however, a raise in PPFD compensated for NUE of plants under N constraint. The same results were also reported in previous experiments on three genotypes of rocket salad^45^, corn^46^, and rice^47^. Being a complex physiological trait, NUE depends on N availability and the energy provided through photosynthesis to supply the energy required for N uptake^46^. Under the N constraint condition, a negative correlation was detected between N concentration and NUE, however, this negative correlation ameliorated by increase in PPFD. Raise in PPFD under N constraint also increased the correlation of NUE with PI_ABS_ and C:N ratio. Suggesting a role for PPFD on enhancing NUE via improving energy provision through photosynthesis for N uptake and maintenance of C:N balance.

## Materials and methods

### Plant materials and growth conditions

The plant experiments were performed in accordance with relevant guidelines and regulations. Rooted cuttings (9 cm long with three leaves) of chrysanthemum (*Chrysanthemum morifolium* cv. Katinka) were sown in pots (14 × 10 cm) containing a mixture of cocopeat and perlite (2:1, v/v), then placed in four growth chambers (1 m × 1 m × 1 m) equipped with a fixed combination of red and blue LEDs with wavelength peaks at 660 ± 10 nm for red and 460 ± 10 nm for blue LEDs. Red and blue LEDs (70:30) were used because they are the main light spectra for photosynthesis and growth of chrysanthemum^13^. To provide different PPFDs, including 75, 150, 300, and 600 µmol m^−2^ s^−1^ and also to limit the production of heat by the light sources, LED light panels (provided by Iran Grow Light Co, Iran) were used. Light spectra and PPFDs were monitored using a Sekonic light meter (Sekonic C-7000, Japan). All plants were grown under the same climatic conditions, i.e. day/night temperature of 25/20 ± 2 °C, 50 ± 5% relative humidity (RH), and photoperiod 12/12 h light/ dark cycles. The nutrient solutions of different N levels were modified based on a full-strength Hoagland and Arnon solution and were applied to each pot three times per week (Table 1). The full-strength Hoagland solution, containing 15 mM N L^−1^ was the control solution and Hoagland and Arnon solutions with modified N concentration (5 and 10 mM N L^−1^) were used to apply N limitation.

**Table 1:** The composition of nutrient solutions of different N levels were modified based on a full-strength Hoagland solution.

Element	Full strength Hoagland solution (g/L)	Modified Hoagland solution containing 10 mL nitrogen (g/L)	Modified Hoagland solution containing 5 ml nitrogen (g/L)
KNO_3_	101.1	–	–
CaCl_2_	–	–	110.9
K_2_SO_4_	–	87.1	87.1
Ca(NO_3_)_2_⋅4H_2_O	236.1	236.1	236.1
MgSO_4_⋅7H_2_O	246.5	246.5	246.5
KH_2_PO_4_	136.1	136.1	136.1
H_3_BO_3_	2.86	2.86	2.86
MnCl_2_⋅4H_2_O	1.81	1.81	1.81
CuSO_4_⋅5H_2_O	0.08	0.08	0.08
Na_2_MoO_2_⋅2H_2_O	0.12	0.12	0.12
H_2_MoO_2_⋅H_2_O	0.09	0.09	0.09
ZnSO_4_⋅5H_2_O	0.22	0.22	0.22
FeNaEDTA	4.04	4.04	4.04

The concentration of 15 mM N L^−1^ (control) was achieved via the full-strength Hoagland and Arnon solution. Concentrations of 10 and 5 mM N L^−1^ were achieved by total or partial substitution of KNO_3_ and Ca(NO_3_)_2_ as sources of nitrogen in the solution. Potassium and calcium levels were equalized across treatments by adding K_2_SO_4_ and CaCl_2_ into the solutions that had N limitations (Table 1)^48^.

### Chlorophyll fluorescence analysis

Fast induction of fluorescence transient (the so-called OJIP protocol) was performed on fully developed, mature leaves of plants after 45 days of growth. The samples were dark-adapted for 20 min. A Fluorpen FP 100-MAX (Photon Systems Instruments, Drasov, Czech Republic) was used for measuring the OJIP transients. Different biophysical and phenomenological parameters related to PSII status^49^ were investigated by JIP-test according to the protocol described by^50^.

The maximum quantum yield of PSII (F_v_/F_M_) and Performance index per absorbed light (PI_ABS_) was calculated using the Eqs. (1) and (2), respectively:

1 $${\text{F}}_{{\text{v}}} /{\text{F}}_{{\text{M}}} = \, ({\text{F}}_{{\text{M}}} {-}{\text{F}}_{0} )/{\text{F}}_{{\text{M}}}$$

2 $$\left( {{\text{RC}}/{\text{ABS}}} \right) \, \times \, \left( {\upphi {\text{P}}_{0} /\left( {{1 }{-} \, \upphi {\text{P}}_{0} } \right)} \right) \, \times \, \left( {\uppsi_{0} /\left( {{1 } - \, \uppsi_{0} } \right)} \right)$$

### Determination of chlorophyll and nitrogen content

Total leaf nitrogen content was determined using 1 g samples of leaf tissue, based on the Kjeldahl method after 98% H_2_SO_4_ hot digestion. For measuring the chlorophyll content, Leaves were measured by using a SPAD-502 (Konica Minolta Corp., Solna, Sweden). This instrument determined the chlorophyll content in leaves non-destructively by considering leaf transmittance in red and near-infrared light spectra. three leaves were analyzed each time and three points were recorded per replicate leaf and were averaged^51^.

### Determination of stomatal traits

Stomatal morphological parameters including stomatal length, stomatal width together with pore length, and aperture were measured on the young fully developed leaves. A section of the leaf midway between the tip and the base and equal distance from each edge were used for microscopic analysis. The abaxial surface of young developed leaves was coated by a thin layer of nail polish. The dry polish sample along with the adhered sticky tape was mounted on microscope slides, and stomatal morphological details were investigated under a light microscope. Images were taken by Omax top-view software version 3.5 and further analyzed using ImageJ software (U.S. National Institutes of Health, Bethesda, MD; https://imageJ.nih.gov/ij/) to record stomatal length, stomatal width, pore length, and aperture^52^.

Stomatal conductance (g_s_) was investigated according to the method described by Fanourakis et al.^53^. In this calculation, stomatal pore depth was considered to be equal to the guard-cell width (i.e., stomatal width/2), assuming guard cells inflate to a circular cross-section. A 100 magnification was used to assess stomatal density. The number of stomata was counted on three randomly chosen areas of the same leaf from which stomatal size measurements were taken. Analysis was done on 1 mm^2^ of the middle of the leaf on both sides of the main vein^52,53^. Calculation of g_s_ was done based on the following equation:

3 $$\mathrm{gs}=\frac{\left(\text{diffusion} \; \text{coefficient}\right)\times \left(\text{stomatal} \; \text{density}\right)\times \left(\uppi \times \text{pore} \; \text{apperture} \div 2 \times \text{ pore} \; \text{length } \div 2\right)}{\left(\text{molar} \; \text{volume} \; \text{of} \; \text{air}\right) \times \left[ \left(\text{pore} \; \text{depth}\right)+ \sqrt{(\text{pore} \; \text{apperture } \div 2 \times \text{pore} \; \text{length } \div 2)}\right]}$$

### Morphological and growth measurements

For biomass determination, the growing substrate was washed from the roots, and the plants were divided into flower, leaves, stem, and roots. The samples were weighed to determine their fresh weight (FW) and then dried in an oven at 60 °C for 72 h to reach a constant dry weight (DW).

### Water use efficiency (WUE)

To determine irrigation water use efficiency (WUE), the amount of water used during the growth period was recorded and after measuring the dry weight of the flowers, it was calculated with the following equation described by Karam et al.^54^.

4 $$\mathrm{WUE}=\frac{\text{Flower} \; \text{dry} \;\text{weight}}{\text{Consumed} \; \text{water}}$$

### Nitrogen use efficiency (NUE)

Nitrogen use efficiency (NUE) was calculated according to the equation below^7,55,56^:

5 $$\mathrm{NUE}=\frac{\text{Flower} \; \text{dry} \; \text{weight}}{\text{N}\; \text{supply}}$$

### Measurements of carbohydrates

Determination of leaf soluble sugar was determined by the anthrone method using glucose as the standard^57^. Fresh leaves (0.2 g) were extracted in 80% ethanol at 80 °C for 60 min. Then 3 mL of anthrone solution (150 mg anthrone in 100 mL 72% Sulfuric acid) was added to 0.1 mL of alcoholic extract. This mixture was placed in a water bath at 100 °C for 10 min and then cooled in an ice bath and the absorbance was spectrophotometrically recorded at 625 nm. The remaining solid part after extraction of soluble sugars was used to extract starch in 52% perchloric acid. Starch concentrations were determined by anthrone and were spectrophotometrically recorded at 630 nm as described by McCready^57^.

### Statistical analysis

The results represented the average mean values of six replications for each treatment. The data were analyzed using SAS software (version 9.4). The two-way analysis of variance (ANOVA) was performed to find the significant differences (p ≤ 0.05) among treatments. Further, the Duncan multiple comparisons test was performed to compare the means. For analyzing chlorophyll fluorescence parameters, obtained data were subjected to two-way ANOVA, and for mean comparison, the Tukey multiple comparison tests were used. For stomatal characteristics, data obtained from one leaf were considered not independent, and for mean comparison, one-way ANOVA, as well as Tukey multiple comparison tests, were used.

## Supplementary Information

Supplementary Information.

## Data Availability

The datasets generated during and/or analyzed during the current study are available from the corresponding author on reasonable request.
