# Supplementary material for: Elevated light intensity compensates for nitrogen deficiency during chrysanthemum growth by improving water and nitrogen use efficiency
Source: Sci Rep. 2022 Jun 15;12:10002. doi: 10.1038/s41598-022-14163-4 (PMC9200816; doi:10.1038/s41598-022-14163-4)
Supplement: Supplementary file 1 — Supplementary Information. [file 41598_2022_14163_MOESM1_ESM.docx]

**Supplemental Figures**

**
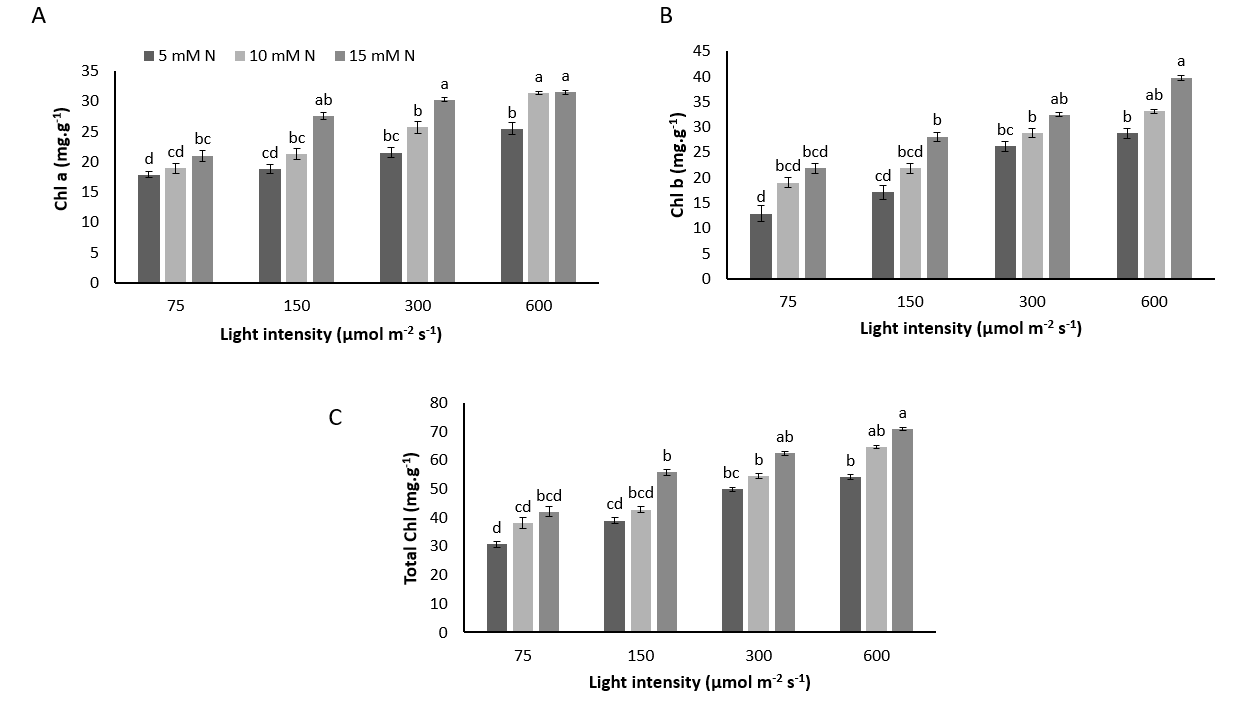
**

Supplementary figure 1 Change in chlorophyll content (including Chl a [A], Chl b [B] and Total Chl [C]) of chrysanthemum plants in response to four light intensity (75, 150, 300 and 600 µmol m^-2^ s^-1^) and three nitrogen concentrations (5, 10 and 15 mM N). Values are the means of six replicates and bars indicate means ± SEM. Within each graph, interactive effects of light intensity and nitrogen concentration are shown. Different letters (a–d) denote a significant difference between treatments (P < 0.05).

Supplementary figure 2 Correlation among different parameters in chrysanthemum plants grown under nitrogen constraint (5 mM N) and high PPFD (600 µmol m^-2^ s^-1^).

Supplementary figure 3 Correlation among different parameters in chrysanthemum plants grown under nitrogen constraint (5 mM N) and low PPFD (75 µmol m^-2^ s^-1^).

Supplementary figure 4 Light curve (LC) of young (Y) and old (O) leaves of chrysanthemum plants in response to four light intensity (75, 150, 300 and 600 µmol m^-2^ s ^-1^) and three nitrogen concentrations (5, 10 and 15 mM N). Values are the means of six replicates and bars indicate means ± SEM. Within each graph, interactive effects of light intensity and nitrogen concentration are shown


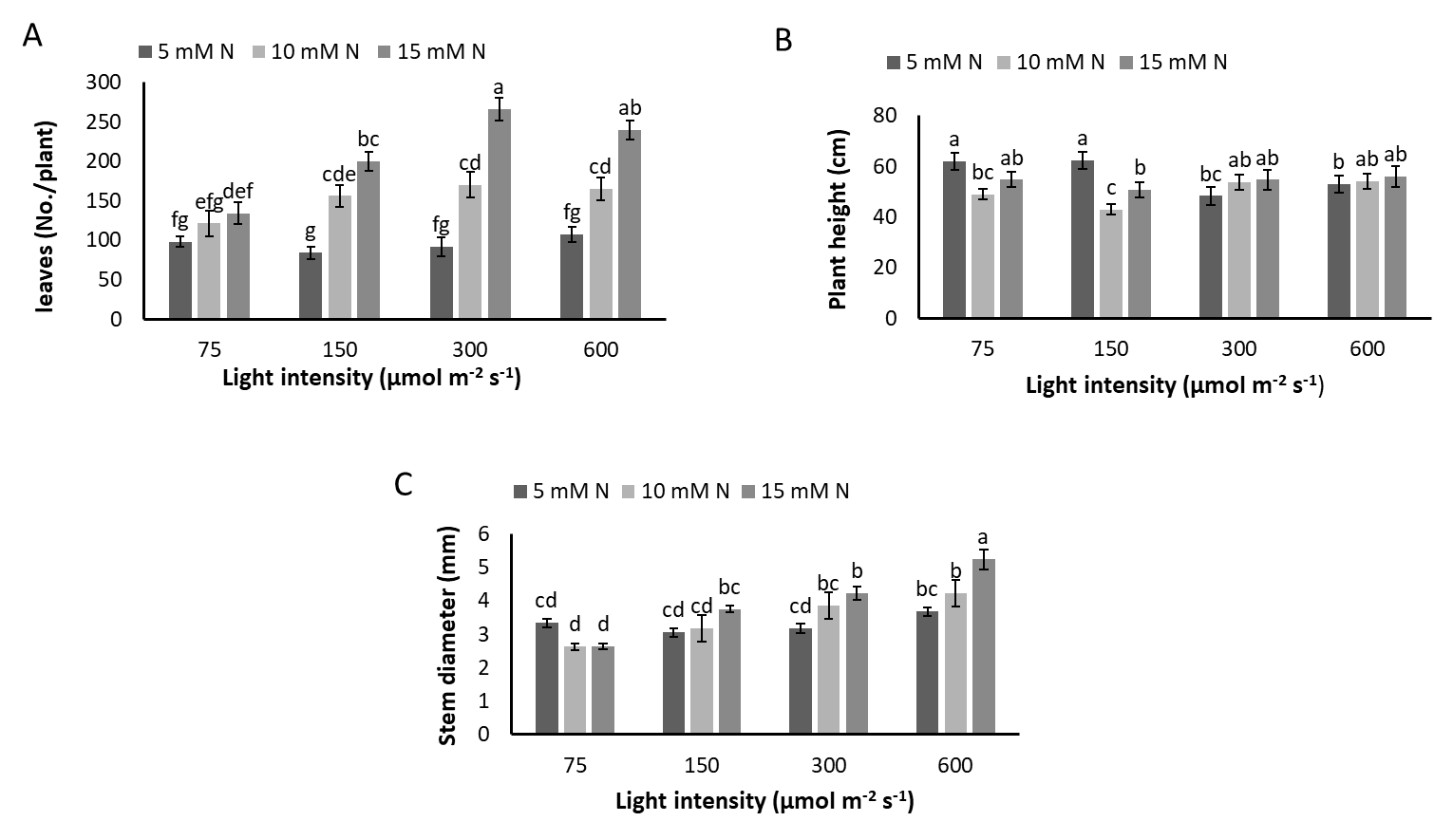


Supplementary figure5 Number of leaves (A), plant height (B) and stem diameter (C) of chrysanthemum plants grown under different light intensits (75, 150, 300, and 600 µmol m^-2^ s ^-1^) and nitrogen (N) concentrations (5,10, and 15 mM N) at 70 days of cultivation. Vertical bars are means ± SD. Within each graph, interactive effects of light intensity and nitrogen concentration are shown. Different letters (a–d) denote a significant difference between treaments (P < 0.05).


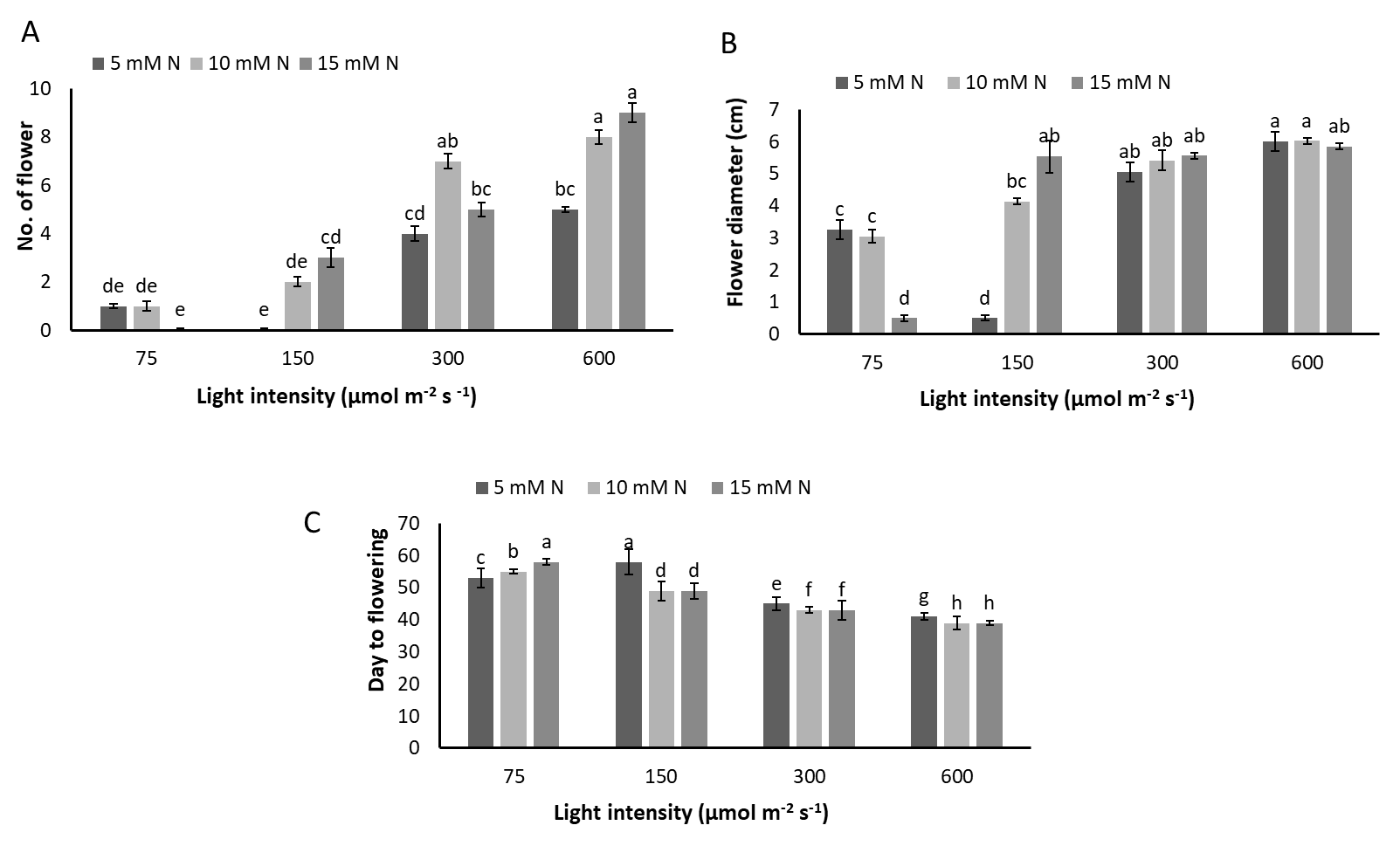


Supplementary figure 6 Number of flowers (A), flower diameter (B) and day to flowering (C) of chrysanthemum grown under different light intensits (75, 150, 300, and 600 µmol m^-2^ s ^-1^) and nitrogen (N) concentrations (5, 10, and 15 mM N) at 70 day of cultivation. Vertical bars are means ± SD. Within each graph, interactive effects of light intensity and nitrogen concentration are shown. Different letters (a–d) denote a significant difference between treaments (P < 0.05).
